# Influence of composite resin type and curing method on the fracture resistance of class II restorations: An in vitro comparison

**DOI:** 10.34172/joddd.025.43966

**Published:** 2025-12-31

**Authors:** Gianella Haydee Vidal-Castillo, Fredy Hugo Cruzado-Oliva, Luis Felipe Alarco-La Rosa

**Affiliations:** Department of Stomatology, Faculty of Stomatology, Universidad Nacional de Trujillo, Trujillo, Peru

**Keywords:** Composite resin, Fracture resistance, Ormocer, Polymerization

## Abstract

**Background.:**

The selection of the restorative material and the use of inadequate curing protocols can affect the mechanical performance of composite resin restorations. This in vitro study aimed to determine the influence of restorative material type and curing method on the fracture resistance of restorations with proximal involvement.

**Methods.:**

A total of 120 extracted human molars were selected, and Cl II cavities (OM) were prepared, maintaining consistent dimensions across all teeth. The samples were randomly divided into two experimental groups: teeth restored with a nanohybrid composite resin (Filtek Z350XT) and teeth restored with Ormocer-based composite resin (Admira Fusion). Each group was further subdivided according to the curing method: pre-curing, co-curing, and simultaneous curing. In addition, two control groups were included: a negative control consisting of prepared but unrestored teeth, and a positive control consisting of intact, sound teeth. Fracture resistance was evaluated using a universal testing machine. Data were analyzed using two-way ANOVA and post hoc Tukey tests (α=0.05).

**Results.:**

Two-way ANOVA revealed significant effects of both the composite resin type (F=1750.4, *P*<0.001) and the curing method (F=210.6, *P*<0.001) on fracture resistance, with a significant interaction between the factors (*P*=0.002). The Ormocer-based composite resin exhibited higher fracture resistance than the nanohybrid composite resin across all curing methods. Within each composite type, the simultaneous curing method showed the highest fracture resistance, followed by co-curing and pre-curing.

**Conclusion.:**

The fracture resistance of Cl II composite resin restorations was significantly influenced by both the composite formulation and the curing protocol.

## Graphical Abstract

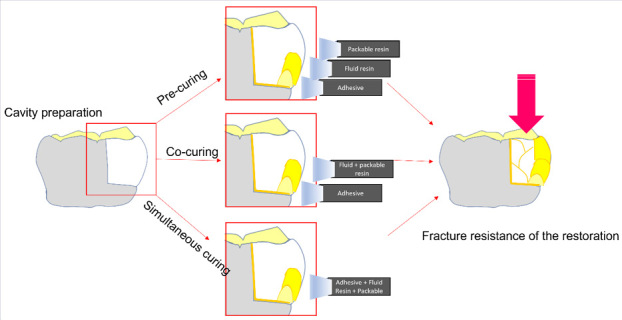

## Introduction

 Since their emergence in the 1960s, composite resins have undergone significant developments.^1^ Improvements in their physicochemical properties and aesthetic appearance have made them the most widely used materials in restorative dentistry.^2,3^ However, they still face several challenges, such as polymerization shrinkage, porosity, water absorption, micro and nanoleakage, and microbial colonization, which cause deterioration of the tooth‒restoration interface.^4-6^

 Moreover, Cl II restorations present inherent challenges, including difficulties in achieving proper technique, limited visibility, and difficult access, which increase the risk of poor marginal sealing. Additionally, the impact on the marginal ridge, high stresses in the isthmus area, and the buckling effect contribute to a higher risk of fracture.^7,8^

 To address these issues, various restorative techniques, low-viscosity composite resins, and curing methods have been introduced, with significant improvements.

 The combination of two techniques, sandwich and centripetal reconstruction, has been suggested. The sandwich technique, described by McLean and Wilson in 1977, involves using a more flowable glass ionomer as a substitute for dentin, which is cured before adding layers of packable composite resin to replace the enamel. This technique can be open or closed, depending on whether the flowable material is exposed to the oral environment. In Cl II restorations, the open technique is used, which is sensitive to moisture.^9^ A decade later, in 1987, Hassan and other researchers proposed the centripetal reconstruction technique, which replaces lost dental structure from the periphery to the center of the cavity by placing a thin layer of packable composite resin toward the matrix band and curing it. Restoration then continues as if it were a Cl I cavity.^10^ A significant problem with Cl II restorations using packable composite resin is inadequate adaptation due to the limited enamel in the cervical proximal area, leading to small gaps.^5^

 This issue was addressed by introducing low-viscosity composite resins as cavity liners beneath the packable composite resin. In the late 1990s, the use of low-viscosity composite resins as liners was introduced, as they serve as a resilient layer between the restoration and the tooth, helping to distribute stresses evenly during polymerization and adapting better to the cavity, thereby reducing microleakage and porosity.^11^

 To further mitigate the issues presented by packable composite resins, various curing methods were introduced: pre-curing, co-curing, and simultaneous curing. In the pre-curing method, also known as Bichacho, the materials involved in the restoration (adhesive, flowable composite resin, and packable composite resin) are photopolymerized separately.^8,12^

 The co-curing method, or snowplow technique introduced by Jackson and Morgan in 2000, proposes individual curing of the adhesive system after its application, followed by co-curing of the increments of flowable and packable composite resins. Placing the packable composite resin over a thin layer of flowable composite resin results in extrusion of the latter out of the cavity, improving marginal sealing.^13^

 Finally, the simultaneous curing method, or injection molding method, differs from the previous methods in that the adhesive, flowable composite resin, and packable composite resin are applied one by one but not photopolymerized separatelyinsteader, all three layers are cured simultaneously.^8,14^

 As previously mentioned, various materials, techniques, and methods have been proposed to address the challenges of Cl II restorations. However, to date, numerous studies have evaluated microleakage and marginal adaptation,^5,9,12^ but few have focused on mechanical resistance. Therefore, the present study aimed to determine the influence of the type of restorative material—Ormocer and nanohybrid—and the curing method (pre-curing, co-curing, and simultaneous curing) on ​​the fracture resistance of Cl II restorations.

## Methods

###  Study Environment

 This study was conducted at the Faculty of Dentistry of the Universidad Nacional de Trujillo, Peru. The study protocol was reviewed and approved by the Permanent Research Committee of the Department of Dentistry of the Universidad Nacional de Trujillo, Peru. The procedures and logistics were approved by the Research Ethics Committee of the same faculty, with the approval code P.I.B. EST. – 025 – 2024.

###  Study Design

 This research was an experimental, cross-sectional, comparative, in vitro study, where the study variables included the curing methods (pre-curing, co-curing, and simultaneous curing) and the restorative material (Ormocer-based composite resin and nanohybrid composite resin), in addition to evaluating a positive and negative control group; the former being a healthy tooth and the latter a prepared Cl II cavity in a tooth without restoration.

###  Sample Selection and Calculation

 A total of 120 healthy molar teeth, free of cracks, fractures, restorations, or structural defects, were collected. These teeth were extracted for orthodontic or prosthetic reasons and donated from private clinics. The sample size was 15 teeth per study subgroup, calculated using the formula for comparing two independent means, with α = 0.10 and β = 0.13. The teeth were randomly distributed into experimental and control groups as follows:

 Nanohybrid composite restorations: Teeth restored with packable composite resin Z350XT (3M^TM^ Filtek^TM^ Supreme, USA) and flowable composite resin Z350XT (3M^TM^ Filtek^TM^ Flowable, USA), subdivided according to the curing method: pre-curing, co-curing, and simultaneous curing.

 Ormocer composite restorations: Teeth restored with packable composite resin Admira Fusion (VOCO, GmbH Cuxhaven, Germany) and flowable composite resin Admira Fusion (VOCO, GmbH Cuxhaven, Germany), subdivided according to the curing method: pre-curing, co-curing, and simultaneous curing.

 Negative control: Teeth with preparation but no restoration

 Positive control: Intact teeth

###  Cleaning and Preparation of Teeth

 The teeth were cleaned with curettes (Gracey, Hu-Friedy, USA) and ultrasound (UDS-A LED, Woodpecker, China) to remove residual soft and hard tissues. They were polished with a mixture of pumice and water using a prophylaxis brush (WH35-01F, Woodpecker, China) and a low-speed micromotor (EX–203C, NSK, Tokyo, Japan). The teeth were stored in saline solution at room temperature for no more than three months after extraction. Subsequently, the root portions of all teeth were embedded in acrylic resin, ensuring parallelism with the base, and the periodontal ligament was simulated using silicone around the roots.

###  Cavity Preparation

**Figure 1:**
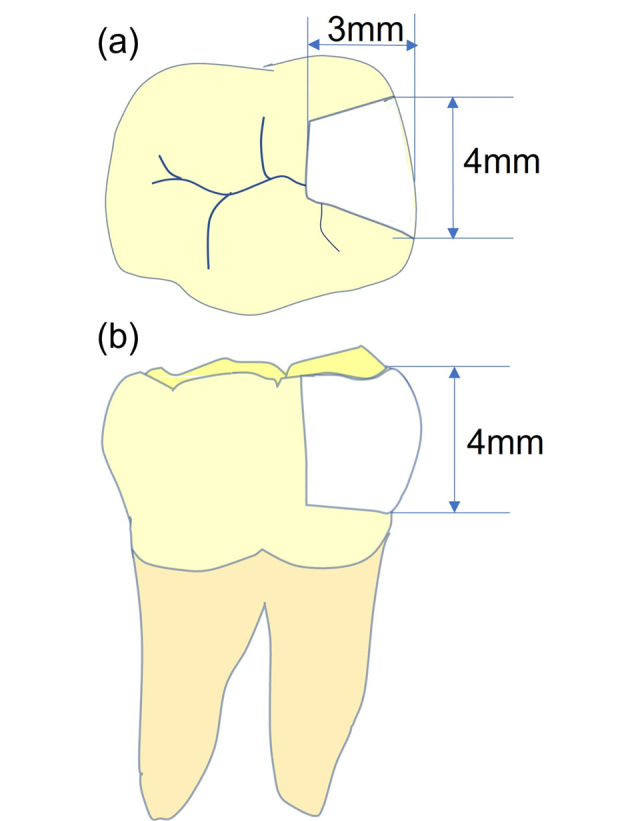
Schematic representation of Cl II cavity preparation (OM). (a) Occlusal view. (b) Sagittal section view

 All cavity preparations (Cl II mesio-occlusal) and restorations were performed by the same operator using a high-speed handpiece (Pana-Max2 B2, NSK, Tochigi, Japan) with cooling, employing a round diamond bur (801-014M, MDT, Israel) and flat-end cylindrical bur (111-012M, MDT, Israel), which were changed after every five cavity preparation procedures. The dimensions were standardized at 4 mm deep, 4 mm wide vestibulo-lingually, and 3 mm wide mesiodistally, with a standard deviation of ± 0.2 mm, calibrated using a periodontal probe (North Carolina, Hu-Friedy, USA) (Figure 1).

###  Cavity Conditioning

 The cavities in the study groups were etched with 37% phosphoric acid gel (Condac37, FGM Dental Group, Brazil) for 20 s on enamel and 15 s on dentin, then rinsed for 15 s and dried with gauze for 7 s without desiccating the dentin.

###  Cavity Restoration

 The experimental groups were restored using packable nanohybrid composite resin Z350XT (3M^TM^ Filtek^TM^ Supreme, USA) and flowable composite resin Z350XT (3M^TM^ Filtek^TM^ Flowable, USA), as well as packable Ormocer-based composite resin Admira Fusion (VOCO, GmbH Cuxhaven, Germany) and Admira Fusion Flow flowable composite resin (VOCO, GmbH Cuxhaven, Germany).

 All occlusoproximal cavities of the study groups were fitted with a circumferential metal band (7 × 0.05 mm [Maquira Dental Group, Paraná, Brazil] and restored using the centripetal technique, filling the vertical cavity on the mesial side first, followed by the remaining occlusal cavity using an oblique incremental technique. For the vertical component, three different photopolymerization methods were employed: the pre-curing or Bichacho method (classic method), the co-curing or snowplow method, and the simultaneous curing or injection molding method.

 In the Bichacho method, an eighth-generation adhesive (All-Bond Universal, Bisco, Schaumburg, USA) was applied to all cavity walls with a disposable brush using a brushing motion for 10 s. The solvent was evaporated with a gentle air stream for 5 s, and the adhesive was light-cured with an LED curing unit (VALO Cordless, Ultradent, Utah, USA) at a wavelength of 395–480 nm and a light intensity of 1000 mW/cm^2^ for 20 s. A layer of flowable composite resin (≈0.5 mm high × 1 mm thick) was then applied over the adhesive layer and photopolymerized for 20 s. Finally, the packable composite resin was applied in increments (≈2 mm high × 1 mm thick) and photopolymerized for 20 s, completing the restoration of the proximal wall.

 In the snowplow method, the adhesive was applied similarly to the previous method. A layer of flowable composite resin (≈0.5 mm high × 1 mm thick) was applied over the adhesive layer, followed by the packable composite resin (≈1 mm high × 1 mm thick) applied over the non-polymerized layer. After condensation, the excess was removed, and both layers were polymerized together for 20 s. Finally, the packable composite resin was applied in increments (≈2 mm high × 1 mm thick) and photopolymerized for 20 s, completing the restoration of the proximal wall.

 The injection molding method involved applying the eighth-generation adhesive (All-Bond Universal, Bisco, Schaumburg, USA), the flowable composite resin (≈0.5 mm high × 1 mm thick), and the packable composite resin (≈1 mm high × 1 mm thick). After condensation, the excess was removed, and all three materials were polymerized together for 20 s. Finally, the packable composite resin was applied in increments (≈2 mm high × 1 mm thick) and photopolymerized for 20 s, completing the restoration of the proximal wall.

**Figure 2:**
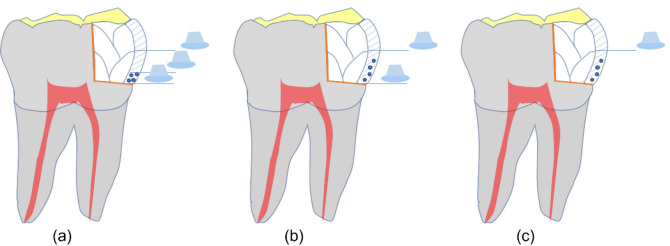
Schematic representation of the restoration with the curing methods. (a) Pre-curing. (b) Co-curing. (c) Simultaneous curing

 The remaining occlusal cavity was restored using an oblique incremental technique of ≈2 mm until the occlusal anatomy of the tooth was restored, with 20 s of photopolymerization between each layer. After completing the restoration, finishing and polishing were performed. Finishing was performed with diamond finishing burs (257-025XF, MDT, Israel), and polishing was performed with resin wheel polishers (DIATECH ShapeGuard, Coltene, Switzerland) (Figure 2).

 This study used the composite resin materials in Table 1. Both flowable and packable composite resins were used, given the need to apply the three photopolymerization methods to both types. Combinations of paste‒flowable from the same manufacturer were employed, and two combinations of current materials were selected.

**Table 1:** Materials used and their chemical composition

**Composite name**	**Composite type**	**Matrix**	**Filler Content / Filler Size (Volume)**	**Manufacturer **
Filtek^TM^ Z350 XT Restorative Universal	Nanohybrid	Bis-GMA, UDMA, TEGDMA, Bis-EMA resins	4-11 nm non-agglomerated/non-aggregated zirconium filling and zirconium cluster/aggregate silica filling, 63.3%.	3M ESPE, St. Paul, MN, USA.
Filtek^TM^ Z350 XT Flowable	Nanohybrid	Bis-GMA, UDMA, TEGDMA, Bis-EMA resins	Silica nanofiller, zirconium nanofiller and zirconium/silica nanocluster 55.5%.	3M ESPE, St. Paul, MN, USA.
Admira Fusion	Ormocer	Silica oxide	Silicon oxide, glass ceramic filler 74%.	VOCO, Cuxhaven, Germany, ceramics (Ormocer)
Admira Fusion Flow	Ormocer	Silica oxide	Silicon oxide, glass ceramic filler 60%	VOCO, Cuxhaven, Germany, ceramics (Ormocer)

###  Fracture Resistance Test

**Figure 3:**
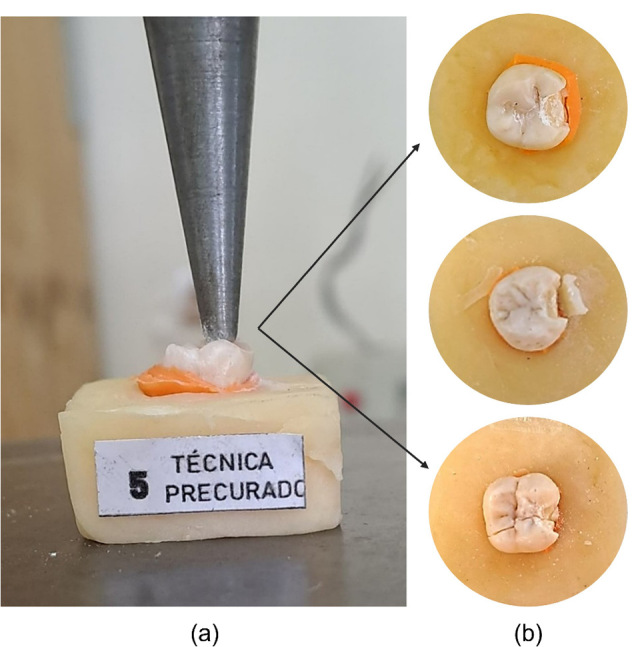
Evaluation of fracture resistance. (a) Indenter of the universal machine. (b) Fracture modes of the restoration and/or tooth

 Fracture resistance was evaluated using a universal testing machine (Tecnotest F 060/EV, Modena, Italy). The samples were subjected to vertical compression with a maximum force of 5 kN and a crosshead speed of 5.0 mm/min, applied in the direction of the restoration (OM) until fracture occurred. The results were recorded using the Eurotronic AD 200 data analysis software (Figure 3).

###  Statistical Analysis

 The data were analyzed using SPSS 22 (Statistical Package for Social Sciences, IBM SPSS, Armonk, NY, USA). Normality assumptions were verified using the Shapiro-Wilk test. Since the data were normally distributed, a two-way ANOVA was performed, followed by post hoc Tukey tests for pairwise comparisons. The confidence interval was set at 95%, and P-values < 0.05 were considered significant.

## Results

 Based on the results presented in Tables 2 and 3, two-way ANOVA revealed significant effects of both composite resin type and curing method, as well as a significant interaction between them (*P* = 0.002).

**Table 2:** Descriptive statistics results for fracture resistance according to composite resin type and curing method

**Composite type**	**Curing method**	**n**	**Mean (N)**	**SD (N)**	**Min – Max (N)**	**CV (%)**
Nanohybrid (Filtek Z350)	Pre - curing	15	1125.3	89.5	940 – 1240	8.0
	Co - curing	15	1222.7	52.2	1140 – 1320	4.3
	Simultaneous curing	15	1280.7	45.9	1220 – 1380	3.6
Ormocer (Admira Fusion)	Pre - curing	15	1406.7	64.4	1270 – 1480	4.6
	Co - curing	15	1630.7	47.0	1560 – 1720	2.9
	Simultaneous curing	15	1657.3	58.1	1580 – 1800	3.5
Controls (not included in ANOVA)	Prepared teeth without restoration	15	457.3	59.9	340 – 550	13.1
	Intact teeth	15	2167.3	78.4	2030 – 2270	3.6

SD: Standard deviation CV: Coefficient of variation

**Table 3:** Two-way ANOVA results for fracture resistance according to composite resin type and curing method

**Two – way ANOVA summary Source of variation**	**df**	**F**	***P*** **-value**	**Interpretation**
Composite type	1	1750.4	< 0.001	Significant effect
Curing method	2	210.6	< 0.001	Significant effect
Interaction (type x method)	2	12.8	0.002	Significant interaction
Error	84	-------	-------	-------

 According to Table 4, post hoc Tukey tests indicated that, within each composite resin type, the simultaneous and co-curing methods resulted in significantly higher fracture resistance compared with pre-curing. Additionally, Ormocer-based restorations consistently exhibited higher fracture resistance than nanohybrid restorations across all curing protocols (*P* < 0.001).

**Table 4:** Post hoc Tukey comparisons for fracture resistance according to composite resin type and curing method

**Group (I)**	**Group (J)**	**Mean difference (I-J)**	**SE**	**95% IC**		***P***
				**Lower bound**	**Upper bound**	
Nanohybrid Pre - curing	Nanohybrid Co - curing	-97.33*	23.21	-169.03	-25.64	= 0.001
	Nanohybrid Simultaneous curing	-155.33*	23.21	-227.03	-83.64	< 0.001
	Ormocer Pre - curing	-281.33*	23.21	-353.03	-209.64	< 0.001
	Ormocer Co - curing	-505.33*	23.21	-577.03	-433.64	< 0.001
	Ormocer Simultaneous curing	-532.00*	23.21	-603.69	-460.31	< 0.001
Nanohybrid Co - curing	Nanohybrid Simultaneous curing	-58.00	23.21	-129.69	13.69	= 0.207
	Ormocer Pre - curing	-184.00*	23.21	-255.69	-112.31	< 0.001
	Ormocer Co - curing	-408.00*	23.21	-479.69	-336.31	< 0.001
	Ormocer Simultaneous curing	-434.67*	23.21	-506.36	-362.97	< 0.001
Nanohybrid Simultaneous curing	Ormocer Pre - curing	-126.00*	23.21	-197.69	-54.31	< 0.001
	Ormocer Co - curing	-350.00*	23.21	-421.69	-278.31	< 0.001
	Ormocer Simultaneous curing	-376.67*	23.21	-448.36	-304.97	< 0.001
Ormocer Pre - curing	Ormocer Co - curing	-224.00*	23.21	-295.69	-152.31	< 0.001
	Ormocer Simultaneous curing	-250.67*	23.21	-322.36	-178.97	< 0.001
Ormocer Co - curing	Ormocer Simultaneous curing	-26.67	23.21	-98.36	45.03	= 0.944

*Statistically significant SE: Stander Error. IC: Confidence interval

## Discussion

 Dental restoration of Cl II cavities remains a topic of ongoing debate. This is primarily due to polymerization shrinkage during the curing of composite resins, which produces stresses at the tooth‒restoration interface and can lead to microporosities and geometric discontinuities, adversely affecting the resistance of the restorations. Therefore, this study proposed the use of a low-viscosity composite resin due to its superior adaptability to the cavity, as supported by the literature.^9,15^ Additionally, the centripetal restoration technique was chosen, as studies demonstrate that it has a higher degree of microhardness^8^ and promising clinical performance.^16^

 Given the benefits provided by the combination of two materials with different viscosities and the centripetal technique,^17^ we aimed to analyze the best clinical outcomes with different curing methods: pre-curing or bichacho, co-curing or snowplow, and simultaneous curing or injection molding, where the difference between them lies in the sequence of polymerization of the materials involved: adhesive, flowable composite resin, and packable composite resin.

 This in vitro study demonstrated that restorations with Ormocer (Admira Fusion Flow + Admira Fusion), regardless of the curing method, exhibited greater fracture resistance than those with nanohybrid composite resins (Filtek^TM^ Z350 XT Flowable + Filtek^TM^ Z350 XT Universal Restorative), and were only surpassed by intact healthy teeth. This finding aligns with existing literature, emphasizing that native dental structure remains the best barrier against occlusal forces, contrasting with teeth that have structural loss and no restoration.^18,19^ These findings coincide with the study by Mărgărit et al.,^19^ which found that molars restored with Ormocer had the highest fracture resistance compared to nanofilled and microhybrid composite resin groups. Similarly, Gunwal et al.^18^ observed that in premolars with MOD cavities, the fracture resistance was significantly greater with Ormocer (1082 N) than with nanohybrid composite resin (778 N).

 The superior performance of Admira Fusion, based on Ormocer, compared to Filtek^TM^ Z350 XT Universal Restorative, based on methacrylate, likely reflects fundamental differences in their chemical composition. Ormocer compounds form a highly cross-linked inorganic‒organic siloxane network, with a higher degree of conversion and lower volumetric shrinkage than conventional methacrylate resins.^20^ In contrast, Filtek^TM^ Z350 XT Universal Restorative contains a significant fraction of the diluent TEGDMA, which increases its volumetric shrinkage and has a very low conversion rate.^21^ These material properties explain why restorations with Admira Fusion showed better fracture resistance; the reduction in shrinkage stress preserves the integrity of the dental structure under load, while the ceramic fillers in Ormocer composite resins confer a high modulus and fracture toughness, distributing crack propagation forces among the different components of the material. This mechanism causes cracks to curve or dissipate when they interact with these particles, thereby limiting their growth and improving the overall strength of the composite resin. According to a two-year follow-up clinical study, bulk-fill Ormocer composite resins exhibit lower polymerization shrinkage and robust physical properties.^20^

 It is also noteworthy that, in terms of microhardness, Ormocer formulations exhibit higher values than nanohybrid and nanoceramic composite resins.^22,23^ In contrast, Rensburg et al.^24^ reported that Filtek^TM^ Z350 XT Universal Restorative exhibited greater microhardness than Admira Fusion; however, this did not translate into worse marginal adaptation or greater leakage, as recent comparative studies found no significant differences in microleakage between both materials. In other words, the greater hardness of the methacrylate-based composite resin did not imply better sealing, and both materials exhibited similar adaptation to the dental substrate.

 On the other hand, the application technique and curing method significantly affected the integrity of restorations. Bonilla et al.^16^ reported no significant difference in fracture resistance between the incremental centripetal technique and the conventional bulk-fill technique, indicating that the geometry of material placement may have less impact than the material composition and curing. In our case, we employed a centripetal technique in all the groups; the results suggest that in this technique, the photopolymerization method was the determining factor.

 This study showed that simultaneous and co-curing methods yield more resistant restorations than the traditional incremental curing (pre-curing) with both restorative materials (Ormocer and methacrylate), which can be explained by the reduction of interfaces between successive layers. By curing the increments together (similar to a bulk-fill method), a more continuous monolithic matrix is formed, minimizing “stratification lines” that could act as weak zones. Additionally, simultaneous curing allows polymerization shrinkage to be distributed more evenly throughout the material’s volume, thereby attenuating stress gradients within each layer. Essentially, reducing photopolymerization cycles minimizes residual shrinkage stress, achieving a more uniform, gradual polymerization that enhances structural integrity. Indeed, Vukelja et al.^25^ observed that the layered application (especially in bulk-fill composite resins) allows for greater light transmission and less shrinkage stress, similar to the injection molding method. In contrast, pre-curing generates multiple successive contractions, increasing internal stress and potentially introducing initial microcracks.^2,26^

 However, in the study by Țuculină et al.^8^, which used finite elements, no differences were found between the three curing methods in terms of maximum stress, deformation, and displacement. This could be explained by the fact that all materials were assumed to be isotropic and did not account for the polymerization shrinkage that occurs during each polymerization process.

 While specific studies on curing methods are scarce, a recent study by Haddad et al.15 reported no significant differences in marginal gap formation between the pre-curing and co-curing methods. These results confirm that the biomechanical performance of restorations is more sensitive to the polymerization protocol.

###  Clinical Implications

 Our data suggest that Cl II restorations using a centripetal technique, with most components cured simultaneously or, at a minimum, the adhesive cured separately and then the composite resins polymerized together, can improve the mechanical resistance of the assembly without compromising sealing quality. This implies that, clinically, the protocol could be simplified (reducing total photopolymerization time) and that shrinkage fatigue could be minimized. For material selection, the results support the use of Ormocer-based composite resins (Admira Fusion) when maximum fracture resistance is required, especially in molars subjected to intense occlusal loads. However, since clinical studies demonstrate good long-term performance of both composite resin types,^26,27^ the final decision should also consider other criteria (aesthetics, handling, and cost). In any case, avoiding excessive pre-curing of each increment and favoring a more integral photopolymerization could be advantageous. The absence of significant differences between simultaneous curing and co-curing indicates flexibility in the protocol; clinicians could choose to cure the adhesive first (practicality) or cure all materials together (efficacy), depending on the situation.

## Study Limitations

 Among the limitations, it should be noted that this is an in vitro study with a single load to failure; cyclic fatigue or long-term aging were not evaluated, leading to uncertainty about the direct correlation with long-term clinical performance. Additionally, fracture pathways or internal microdefects were not analyzed, which would require complementary studies (e.g., microtomography, microscopy) to fully understand the mechanisms. Finally, the results of this study could not be compared with similar studies, as no studies were found in the specialized literature that analyze the mechanical behavior (fracture resistance) of the combination of these three polymerization methods, the selected materials, or a similar restorative technique.

## Conclusion

 Given the limitations of this in vitro study, both the composite resin type and the curing method significantly affected the fracture resistance of Cl II restorations.

 Ormocer-based composite resin (Admira Fusion) exhibited significantly higher fracture resistance than nanohybrid composite resin (Filtek Z350XT) across all curing protocols. Among the curing strategies, simultaneous and co-curing methods resulted in greater fracture resistance than pre-curing, regardless of the composite resin ype.

 These findings suggest that optimizing the curing method and selecting an Ormocer-based composite resin may enhance the mechanical performance of Cl II restorations, with the choice of technique and material ultimately guided by the clinician’s judgment.

## Competing Interests

 The authors declare no conflicts of interest related to this study.

## Ethical Approval

 This study was approved by Ethics committee of Universidad Nacional de Trujillo, Faculty of Stomatology, Ethical code: P.I.B. EST.-025-2024.
